# Prevalence, Antimicrobial Susceptibility Patterns, and Risk Factors Associated with Enterococci among Pediatric Patients at Dessie Referral Hospital, Northeastern Ethiopia

**DOI:** 10.1155/2021/5549847

**Published:** 2021-04-16

**Authors:** Admasu Abera, Mihret Tilahun, Saba Gebremichael Tekele, Melaku Ashagrie Belete

**Affiliations:** ^1^Department of Medical Laboratory Science, Debre Berhan Health Science College, Debre Berhan, Ethiopia; ^2^Department of Medical Laboratory Science, College of Medicine and Health Sciences, Wollo University, Dessie, Ethiopia

## Abstract

**Background:**

*Enterococcus* species, which is previously considered as medically not important, now becomes one of the leading causes of nosocomial infections. Even though it becomes the most public health concern and emerging multidrug-resistant pathogen, there is no enough data in the study area.

**Objective:**

To determine the prevalence, antimicrobial resistance pattern, and associated risk factors of enterococci infection in pediatric patients.

**Methods:**

A hospital-based cross-sectional study was conducted from February to May 2019 at Dessie Referral Hospital, Northeastern Ethiopia. A total of 403 pediatric patients were included in the study, and a pretested structured questionnaire was used to collect sociodemographic and risk factor-related data. Clinical samples such as urine, blood, wound swabs, discharges, and other body fluids were collected aseptically and inoculated on to Bile Esculin Azide Agar, and colony characteristics, Gram stain, catalase, salt, and temperature tolerance tests were employed for bacterial identification. Antimicrobial sensitivity tests were performed using the modified Kirby-Bauer disk diffusion method. Data was entered into SPSS software version 25 and descriptive statistics; bivariate and multivariate logistic regression analyses were performed. In all cases, a *P* value ≤ 0.05 with corresponding 95% confidence interval were considered as statistically significant.

**Result:**

The overall prevalence of enterococci was 2.7% (11/403). Of which, the highest number of enterococci infection was recovered from urine sample (54.5%) followed by blood (27.3%), wound swab (9.1%), and other body fluids (9%). The overall multidrug resistance rate was 54.5%. Higher drug resistance pattern was observed against tetracycline, chloramphenicol, and amoxicillin/clavulanate. Having history of invasive procedure (*P* < 0.001), chronic illness (*P* < 0.001) and previous admission history of the children (*P* < 0.001) were statistically significant associated risk factors for pediatrics enterococci infection.

**Conclusion:**

The prevalence of enterococci from pediatric patients in this study was relatively low compared to other studies. Significant rates of MDR and VRE were identified, and the risk of infection became high when children had a history of different chronic illnesses and history of admission and underwent invasive treatment procedures. Therefore, efforts should be made to prevent enterococci infections and spread of multidrug-resistant enterococci.

## 1. Introduction

Enterococci are members of Gram-positive *Enterococcaceae* family and are nonspore forming, facultatively anaerobic, oxidase, and catalase negative bacteria that occur singly, in pairs or short chains [1]. Moreover, enterococci are known to survive in a range of hostile environments, including growth in the presence of 6.5% NaCl, temperature of 5 to 65°C, pH 4.5 to 10, and hydrolyze esculin in the presence of 40% bile [1, 2]. Enterococci are common indigenous flora of the gastrointestinal tracts and can also normally present in the oral cavity, vagina, oropharynx, and urethra of humans [3].

In fact, enterococci species have low virulence factors. However, under conditions in which the host encountered immune suppression or the integrity of the gastrointestinal or genitourinary tract has been disrupted, they spread to normally sterile sites and cause various infections. These infections include urinary tract infections, wound infection, sepsis, endocarditis, intra-abdominal abscesses, and biliary tract infections [4].

According to Centers for Disease Control and Prevention (CDC) nationwide report, enterococci are one of the leading causes of nosocomial infections worldwide, with healthcare-associated infections occurring more frequently in resource-limited settings than in developed countries [5]. Moreover, the pathogen distribution and antimicrobial resistance patterns in healthcare-associated infections (HAIs) from pediatric groups also indicated 8% of enterococci species [6].

Different risk factors have been reported to be associated with the spread of enterococci infections including concurrent infections, history antibiotic use, surgery, catheterization, longer duration of hospitalization, and underlying immunosuppressing diseases such as HIV, diabetics, and cancer [7].

The emergence and spread of antimicrobial resistance among enterococcus species pose enormous challenges for clinicians, especially in the management of severe infections. The increased prevalence and dissemination of multidrug-resistant enterococcus has narrowed the therapeutic options globally, as the majority of enterococcus isolates exhibit a high level of resistance to ampicillin, penicillin, and vancomycin, which are indeed the most historically useful anti-enterococci antibiotics [8].

In 2018, the national survey for antimicrobial resistance in Europe report indicated that vancomycin-resistant enterococcus species increased significantly from 10.4% in 2014 to 14.9% in 2017 in different countries [9]. Moreover, according to worldwide surveillance summary of drug potency of Gram-positive pathogens, the overall prevalence of vancomycin-resistant enterococci (VRE) in Europe, Asia and Pacific, and Latin and North America ranges between 1% and 9.8% [10].

The prevalence and drug resistance figures are significantly high in developing countries. In Africa and Ethiopia, some reports indicated the overall prevalence of enterococci and antibiotic resistance ranged from 2.2% to 76% and 6.3% to 95.5%, respectively [11–16]. Therefore, efforts should be made to prevent enterococci infections and emergence of multidrug-resistant enterococci. Hence, studying the prevalence, dispersion, and correlation of possible associated risk factors and examining the drug resistance pattern of enterococcus species, particularly in vulnerable target groups such as pediatrics, are important for effective prevention, control, and management of infections. Therefore, the study was aimed at determining the prevalence, antimicrobial susceptibility patterns, and associated risk factors of enterococci among pediatric patients in Dessie Referral Hospital (DRH), Northeastern Ethiopia.

## 2. Method and Materials

### 2.1. Study Design, Area, and Period

A hospital-based cross-sectional study was conducted from February 2019 to May 2019 at Dessie Referral Hospital, South Wollo Zone of Amhara regional state, Northeast Ethiopia. Dessie Referral Hospital is located in Dessie city, 400 km from the capital, Addis Ababa, and 471 km far from Bahir Dar, the capital of the Amhara regional state. The city has one referral hospital, one general hospital, three private general hospitals, five health centers, eight private higher clinics, and one Public Health Research Institute. Dessie Referral Hospital provides emergency, antiretroviral therapy (ART) services, chronic care, surgical, dental, medical, pediatric, gynecologic, obstetric, and other services. The hospital serves patients from all parts of the region which comprises a population of more than 4 million people. Dessie Referral Hospital pediatric services consist of inpatient and outpatient departments. The outpatient pediatric department consists of 15 beds and five rooms, while the inpatient pediatric ward consists of 13 rooms and 49 beds, with an average of about 7 children admitted per day.

### 2.2. Inclusion and Exclusion Criteria

All children younger than 15 years old, attending DRH and who were requested for laboratory investigation during the study period, were included in this study. On the other hand, pediatric patients who received antibiotics within the past 2 weeks were excluded. Moreover, as enterococci are normal flora of some specific sites including respiratory, genital, and gastrointestinal tracts, samples such as sputum, throat swab, stool, and vaginal swabs were excluded from the study.

### 2.3. Sample Size Determination and Sampling Technique

The sample size was determined using a single population proportion formula considering 50% prevalence, marginal error of 5%, and 95%confidence interval = 1.96 by using the following sample size determination formula:

(1) $$\begin{array}{c} n=\frac{z^{2}p\left(1-p\right)}{d^{2}}, \end{array}$$

where *n* is the minimum sample size required, *Za*/2 is the significant value for 95% confidence interval, *P* is the expected prevalence of enterococci infection, and *d* is the margin of error.By including 5% nonresponse rate, a total of 403 children were included in this study using systematic random sampling.

### 2.4. Data and Specimen Collection

Data were collected using a short interview guided by pretested structured questionnaire consisting of the client's sociodemographic, clinical, and risk factor data. Clinical samples were collected from each study participant aseptically. About 5 ml of the blood sample was collected from children and dispensed into blood culture bottle prepared with 25 ml of Tryptic Soya Broth (FL Medical, Italy) aseptically. Ten ml of freshly voided midstream urine specimen was collected using wide mouth, leak-proof, sterile, plastic container under the supervision of the principal investigator and processed within 2 hours of collection. Approximately 5 ml of cerebrospinal fluid (CSF) sample was collected aseptically into sterile tube by lumbar or ventricular puncture performed by a physician and processed within one hour of collection. Wound swab, pus, eye, and ear discharges were obtained using sterile cotton tip applicator stick aseptically. The blood sample was transported with the blood culture broth while all swabs were transported within BHI broth (HiMedia™). The collected specimens were then stored in a cold box and transported to the Department of Microbiology Laboratory, Amhara Public Health Institute (APHI), Dessie Branch. Immediate inoculation had been performed for all specimens on arrival to the laboratory.

### 2.5. Isolation and Identification of Enterococci

Specimens were inoculated on appropriate culture media to isolate the enterococci bacteria. The blood culture bottles were incubated at 37°C and observed after 24 hrs daily for consecutive 5 days for the presence of turbidity, hemolysis, gas formation, or color changes which are evidence of microbial growth. If the culture bottle does not show any growth within 7 days, it was reported as negative. Whenever visible growth appears, the bottle was opened aseptically, a small amount of broth was taken with a sterile loop and subcultured on Bile Esculin Azide agar (BEAA) (Oxoid Ltd., UK). Urine samples were inoculated on BEAA media with a 1 *μ*l calibrated loop and incubated at 37°C for 24 hr. Presence of ≥10^4^ colony forming unit (CFU) per ml of urine with the black colored colony was considered as significant enterococci in urine specimen. Other clinical samples were directly subcultured on BEAA and incubated at 37°C for 24 hr and were checked for growth of very small colony with blackening media. Colony characteristics, Gram staining reaction, catalase, salt tolerance, and temperature tolerance tests were used for identification of enterococci [17].

### 2.6. Antimicrobial Susceptibility Testing

The antimicrobial susceptibility testing of enterococci isolates was performed using the Kirby-Bauer disk diffusion technique as modified by the Clinical and Laboratory Standard Institute (CLSI) in 2019 [18]. From a pure culture, 3-5 selected colonies of bacteria were taken and transferred to a tube containing 5 ml sterile normal saline and mixed gently to make homogenous suspension and the turbidity of the suspension was adjusted comparably to a McFarland 0.5 turbidity standard. A sterile cotton swab was used to streak the plates, and the excess suspension was removed by gentle pressing and rotation of the swab against the inside wall surface of the tube. The swab was then used to distribute the bacteria evenly over the entire surface of Mueller Hinton agar (MHA) (Conda Ltd., USA). The inoculated plates were then left at room temperature to dry for 3-5 minutes, and a set of 6 antibiotic discs were applied on the MHA. Based on availability, CLSI recommendation, and prescription practice of DRH, the test was carried out for the following drugs: erythromycin (E, 15 *μ*g), chloramphenicol (C, 30 *μ*g), tetracycline (TE, 30 *μ*g), ampicillin (AMP, 10 *μ*g), nitrofurantoin (NIT, 30 *μ*g), ciprofloxacin (CIP, 5 *μ*g), penicillin (P, 10 IU) and vancomycin (VA, 30 *μ*g), and amoxicillin-clavulanic acid (AMC, 20/10 *μ*g). All antibiotic discs were from Oxoid Ltd., UK. The plates were then incubated at 37°C for 24 hours. Diameters of the zone of inhibition around the discs were measured using a digital caliper. The interpretation of the results of the antimicrobial susceptibility tests was based on the standardized table supplied by CLSI [18] criteria as sensitive, intermediate, and resistant.

### 2.7. Quality Assurance

Data quality was ensured by using standardized data collection materials. The questionnaire was pretested on 5% of the sample size in the nearby Boru Meda Hospital before the actual study commenced to make sure whether the questionnaire is appropriate and understandable. All the questions in structured questionnaire were prepared in a clear and precise way and translated into local language (Amharic). The collected data were checked daily for completeness. Moreover, all laboratory analyses were performed by maintaining quality control procedures. Standard operating procedures (SOPs) were strictly followed verifying that media meet expiration date and quality control parameters per CLSI guideline. All culture media were prepared following the manufacturers' instructions. Batch of prepared media was checked for pH, performance, and sterility test by incubating samples of the plate at 37°C for 24 hrs, and reagents for Gram stain and biochemical tests were checked using known standard strains of *Enterococcus faecalis* ATCC 29212, *Escherichia coli* ATCC 25922, *Streptococcus pyogenes* ATCC 19615, and *Staphylococcus aureus* ATCC 25923, and cotrimoxazole antibiotic drug was used to check quality of MHA. All reference strains were obtained from Microbiology Laboratory of APHI, Dessie Branch.

### 2.8. Statistical Analysis

Data was entered and analyzed using Statistical Package for Social Sciences (SPSS) version 25.0. (IBM, USA), and descriptive statistics, binary, and multivariate logistic regression were computed. The bivariate analysis using maximum likelihood estimates of the categorical variables was used to determine the association of each variable with the dependent variable. Furthermore, variables with *P* < 0.2 in the bivariate analysis were subjected to multivariate logistic regression to identify the independent predictors of enterococci infections. *P* value < 0.05 with 95% confidence interval was considered statistically significant.

## 3. Result

### 3.1. Sociodemographic Characteristics of Study Participants

A total of 403 study participants were included during the study period. Of these, 188 (46.7%) were males, while 215 (53.3%) were females. The age of the study participants ranged from 15 days to 14 years. The age distribution of participants indicated that majority 159 (39.5%) of the study participants were in the age group of 5 to 9 years followed by 124 (30.8%) belonging to the age group ≤4 years. Slightly majority of the study participants 214 (53.1%) were rural dwellers. Moreover, the clinical data showed that outpatient was the predominant group among the study participants, with a proportion of 67.2%, while the remaining 32.3% were from inpatient (Table 1).

### 3.2. Prevalence of Enterococci Isolates among Pediatric Patients

**Figure 1:**
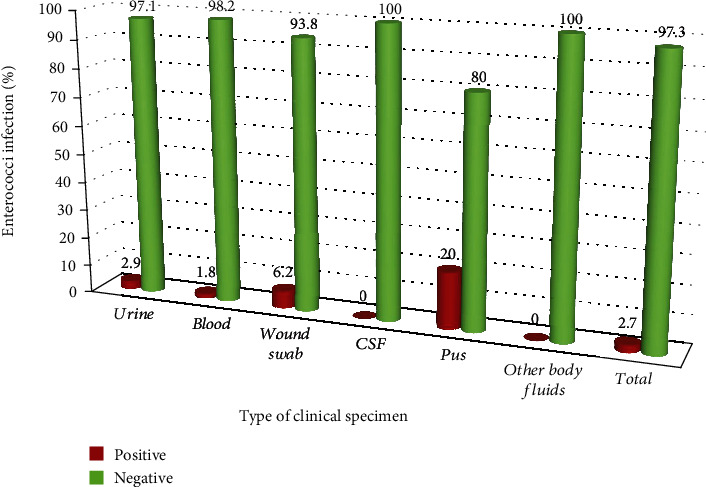
Distribution of enterococci infection in different clinical specimens of pediatric patients at DRH from February to May 2019.

A total of 11 (2.7%) enterococci isolates were isolated from all clinical samples. Seven out of 11 isolates were identified from outpatients. Relatively higher frequency of enterococci infection (5/11) was observed in the age group of ≤4 years compared to the other age groups (Table 2). Moreover, the peak positivity rates were obtained from pus sample (20%) followed by wound swab (6.2%) and urine specimens (2.9%) (Figure 1).

### 3.3. Association of Risk Factors for the Acquisition of Enterococci Infection

In this study, a total of 14 independent variables were considered during the bivariate analysis of risk factors for enterococci infection. In the multivariate analysis, the presence of enterococci infection was significantly associated with having a history of invasive procedure (AOR = 26.91, 95% CI: 4.96, 148), chronic illness (AOR = 16.91, 95% CI: 2.98, 88.08), and history of admission (AOR = 13.73, 95% CI: 3.01, 62.59) with *P* < 0.001 for all (Table 3).

### 3.4. Antimicrobial Susceptibility Pattern of Enterococci

The enterococci isolates showed a higher level of drug resistance to tetracycline (10/11), chloramphenicol (9/11), augmentin (8/11), and erythromycin (7/11), while 5/11 resistance rates were reported for the rest of the antibacterial agents. Moreover, nearly half (5/11) of the isolated enterococci were vancomycin-resistant (Table 4).

### 3.5. Multiple Drug Resistance Pattern of the Isolates

All detected enterococcus isolates (11) were resistant to at least one antimicrobial agent, whereas 9 isolates were resistant to ≥2 antimicrobials. Multidrug resistance (defined as nonsusceptibility to at least one agent in three or more antimicrobial categories) was seen in 6/11 of the enterococci isolates (Table 5).

## 4. Discussion

In the past 1970s and 1980s, enterococci have been changed from being intestinal normal flora of little clinical significance to becoming one of the most common nosocomial pathogens associated with significant morbidity and mortality worldwide [19]. The present study was conducted to determine the prevalence of enterococci infection, evaluates the pattern of drug susceptibility and identify possible risk factors associated with enterococci infection among pediatric patients. The overall prevalence of enterococci in the present study was 2.7% (11/403). This finding was consistent with the previous report of European Center for Disease Control report 1.8% [6], Nepal 3.4% [20], and India 4% [21]. Similar findings were also reported in Ethiopia of Jimma (2.23%) [14], Addis Ababa (2.23% and 1.82%) [22, 23] and Gondar (2.13%) [24]. However, it was lower than the previous reports from Turkey 6.5% [25], India 6% [26], and Gondar 8.9% [16]. The differences with the findings of this study may be attributed to the variation in geographical area, number of samples examined, the extent of time, and method used for sampling.

In this study, enterococci species were most predominantly isolated from urine specimens followed by blood and wound swabs. This was similar to previous reports conducted in Addis Ababa Ethiopia [27], India [28], and Iran [20]. This indicated enterococci infection mostly causes UTI, bacteremia, and wound infections. The most probable reason for the high isolation rate of enterococci from UTI cases might be due to the close proximity of anal opening to the urethra as enterococci reside as commensals in gastrointestinal tract. With this, uropathogens originate from the fecal flora, spread across the perineum, and invade the bladder through the urethral opening. Moreover, urinary catheterization also has a role in some cases and contributed to higher isolation of enterococci from urine specimens.

The present study showed that enterococci isolates had an overall multidrug resistance rate of 54.5%. This finding was in line with a study report from Ethiopia 60% [29]. However, considerably higher in comparison to other study findings from Tunisia, 7.1% [30], and India, 10% [21]. This discrepancy might be due the socioeconomic difference, type of clinical sample used, and age difference of the target study groups. On the contrary, the current finding was lower than a study report from Spain, 80% [31], and India, 82% [32]. Similarly, lower than all studies performed in Ethiopia: Jimma, 89.5%, 80.8% [11, 14], Southern Ethiopia, 82.5% [15] and Gondar, 75% [16]. The variation might be due to differences in clinical sample used and age difference of the target study groups. Moreover, these MDR pathogens call for establishment of targeted antimicrobial resistance (AMR) surveillance system, focused infection prevention and control (IPC) mitigation strategies and introduction of cost-effective diagnostic tools for their routine screening in the hospital settings.

Bacterial drug resistance for the commonly used antibiotics is currently spreading globally. In our study, 5/11 (45%) of the enterococci isolates were resistant to ampicillin, penicillin, vancomycin, ciprofloxacin, and nitrofurantoin. This was in line with the report from India, 52.6% [28], and Jimma Ethiopia, 54.5% [14]. However, our finding was higher than study reports from Ethiopia: Addis Ababa, 6.7% [23], Gondar, 5.5% [12], and Canada, 19% [33]. Besides, our finding was lower than study reports from southern Ethiopia, 100% [34], and Gondar 72% [16]. Moreover, a study conducted in two primary hospitals of southwest Nigeria showed 100% resistant to ampicillin [35]. The highest rate of resistance to ampicillin might be the sign of prolonged irrational usage of the drug in the area.

The rate of 5/11 (45.5%) vancomycin-resistant enterococci (VRE) in the current study has made the scenario worse as it is the preferred choice of drug in the case where this bacterium became resistant to other antibiotics in the area. Even though the result was comparable with the study report undertaken in all age groups in Gondar, 41.5% [16], and Nigeria, 42.9% [35], while it was by far greater than reports of other studies conducted among HIV patients in Ethiopia: Gondar, 5.5% [12], southern Ethiopia, 7.5% [15], Addis Ababa, 6.7% [23], Jimma, 22.7% [11], 6.3% [14], west Iran, 24% [36], Canada, 4% [33], and reports of a nationwide study from Europe, Asia and Pacific, Latin and North America, 1% to 9.8% [10]. The possible reason might be due to the gradual change in the MDR strains, antibiotics selective pressure, differences in drug prescription and usage habits, and socioeconomic status.

The present study revealed that nearly half (45%) of the isolated enterococci showed resistance to ciprofloxacin, which was comparable with reports from Addis Ababa, 53.3% [23]. However, this finding was relatively higher than reports of Dessie, 7% [37], Jimma, 22% [14], Gondar, 33.8% [12], southern Ethiopia, 33.3% [34], Addis Ababa, 14.3% [22], and Canada, 40% [33]. The discrepancy might be due to the significant increment of self-medication and indiscriminate use of ciprofloxacin in the study area.

In this study, higher antimicrobial resistance was seen against commonly used antibiotics including tetracycline (91%), amoxicillin/clavulanic acid (72.7%), chloramphenicol (83%), and erythromycin (66.7%). This finding was higher than reports from southern Ethiopia [34], Jimma [14], Gondar [16], and India [32, 36]. This was the most striking finding of this study which is alarming indicator of heightened prevalence of multidrug resistance. The possible justification for such upsurge in resistance pattern of enterococci species might be the gradual spread of drug resistant strains, especially in developing countries where there is high usage habit, these antibiotics mainly due to their easy availability and low cost. Moreover, these antibacterial drugs are commonly prescribed irrationally by medical practitioners as an empirical treatment options, and self-prescribing of these drugs is also common [38], which in turn causes emergence and spread of drug resistance.

The multivariate analysis of the present study showed that having a history of invasive treatment procedure was significantly associated with pediatrics enterococci infection (*P* < 0.001). This report was in line with a study done in primary hospital of South Korea [39]. The main reason for the survival of enterococci infection in patients undertaking invasive medical procedure/treatment was the fact that foreign devices could be used as a portal of access by disruption of the normal integrity of the body. Moreover, invasive medical procedures could result in displacement of indigenous commensals from their resident sites and easily invade another tissue or organ. Some members of the microbiota, including indigenous commensal enterococci can act as opportunistic pathogens and translocate across the mucosal barrier to cause systemic infection in immune-compromised hosts, increasing the risk of enterococci invasion.

The present study also showed that having a history of chronic illness (*P* < 0.001) and admission or hospitalization (*P* < 0.001) was significantly associated with pediatrics enterococci infection. This report was comparable with a study done among hospitalized patients in Germany [40]. This association is mainly attributed to the effect of chronic illness and hospitalization in diminishing immune status of patients which allows enterococci infection to flourish and cause a clinical illness [41]. Moreover, hospitalization further expose patients, particularly children, to acquire enterococcal infection readily through contamination from the healthcare environment, in which high densities of contaminants found on medical devices (such as blood pressure cuffs, intravenous fluid pumps, or stethoscopes), gowns, bed rails, bedside tables, bed linens, urinals, and bedpans [3].

Species investigation was not performed due to financial issue and shortage of reagents and materials.

## 5. Conclusions

The prevalence of enterococci from pediatric patients in this study was relatively low compared to other studies. The risk of infection became high when children have a history of different chronic illness and history of admission and undergoes invasive treatment procedures. A significant rate of multidrug-resistant enterococci including VRE was identified from clinical samples in the study area. Such occurrence of MDR and VRE showed limited antibiotic treatment options for enterococci infections. Therefore, efforts should be made to prevent nosocomial enterococci infections and spread of multidrug-resistant enterococci. Periodic evaluation of drug susceptibility pattern is also essential for rational and appropriate use of antibiotics. Moreover, appropriate prescription and use of antibiotics and large-scale species identification and genotypic studies at national level are necessary.

## Figures and Tables

**Table 1:** Sociodemographic characteristics of pediatric patient study participants at DRH from February to May 2019.

Demographic characteristics	Frequency in number	Frequency in percent
Age		
≤4	124	30.8%
5-9	159	39.5%
10-14	120	29.8%
Sex		
Male	188	46.7
Female	215	53.3%
Residence		
Urban	189	46.9%
Rural	214	53.1%
Patient setting		
Outpatient	271	67.2%
Inpatient	132	32.8%
Family educational status		
Illiterate	178	44.2%
Read and write	111	27.5%
High school	47	11.7%
Diploma	40	9.9%
First degree and higher	27	6.7%

**Table 2:** Prevalence of enterococci infection among pediatric patients (*n* = 403) at DRH from February to May 2019.

Demographic characteristics	Enterococci infection	
	Positive (%)	Negative (%)
Age		
≤4	5 (45%)	119 (96.0%)
5-9	2 (18.2%)	157 (98.7%)
10-14	4 (36.8%)	116 (96.7%)
Sex		
Male	5 (45.4%)	183 (97.3%)
Female	6 (54.5%)	209 (97.2%)
Residence		
Urban	4 (36.3%)	185 (97.9%)
Rural	7 (63.7%)	207 (96.7%)
Patient setting		
Outpatient	7 (63.7%)	125 (94.7%)
Inpatient	4 (36.3%)	267 (98.5%)
Family educational status		
Illiterate	5 (45.4%)	173 (97.2%)
Only read and write	2 (18.2%)	109 (98.2)
Completed high school	3 (27.3%)	44 (93.6%)
College diploma	1 (9.1%)	39 (97.5)
First degree and higher	0 (0.0%)	27 (100.0%)
Total	11 (2.7%)	392 (97.3%)

**Table 3:** Bivariate and multivariate analysis of associated factors for acquiring enterococci infection among pediatric patients attending DRH from February to May 2019.

Variables (total no.)	Enterococci infection		COR (CI 95%)	*P* value	AOR	*P* value
	Positive (%)	Negative (%)				
Age						
≤4 (124)	5 (4%)	119 (96.0%)	1.21 (0.319-4.65)	0.77	NA	
5-9 (159)	2 (1.3%)	157 (98.7%)	0.360 (0.06-2.05)	0.25		
10-14 (120)	4 (3.3%)	116 (96.7%)	1			
Sex						
Male (188)	5 (2.7%)	183 (97.3%)	1.05 (0.31-3.50)	0.93	NA	
Female (215)	6 (2.8%)	209 (97.2%)	1			
Residence						
Urban (189)	4 (2.1%)	185 (97.9%)	1.56 (0.45-5.42)	0.48	NA	
Rural (214)	7 (3.3%)	207 (96.7%)	1			
Patient setting						
Inpatient (271)	7 (5.3%)	125 (94.7%)	3.73 (1.07-13.00)	0.038	0.33 (0.10-2.13)	0.48
Outpatient (132)	4 (1.5%)	267 (98.5%)	1			
Family educational status						
Illiterate (178)	5 (2.8%)	173 (97.2%)	0.63 (0.12-3.33)	0.59	NA	
Read and write (111)	2 (1.8%)	109 (98.2)	2.35 (0.54-10.25)	0.25		
High school (47)	3 (6.4%)	44 (93.6%)	0.88 (0.10-7.80)	0.91		
Diploma (40)	1 (2.5%)	39 (97.5)	1.02 (0.21-6.37)	0.99		
First degree and higher (27)	0 (0.0%)	27 (100.0%)	1			
History of						
Antibiotic administration	10 (4.3%)	221 (95.7%)	7.81 (0.99-61.66)	0.052	10.17 (0.50-206.3)	0.131
Invasive procedure	5 (17.2%)	24 (82.8%)	28.2 (7.77-102.23)	<0.001	26.91 (4.96-148.99)	<0.001^∗^
Malnutrition	2 (8.3%)	88 (91.7%)	3.73 (0.76-18.35)	0.101	6.31 (0.5-28.09)	0.076
Burn	1 (14.3%)	6 (85.7%)	6.43 (0.70-58.53)	0.260	NA	
Animal contact	3 (6.4%)	44 (93.6%)	2.96 (0.75-11.59)	0.110	0.11 (0.15-0.93)	0.043
Chronic illness	6 (9.2%)	59 (90.8%)	6.77 (2.00-22.91)	0.021	16.21 (2.98-88.08)	<0.001^∗^
Contact with health profess	5 (6.1%)	77 (93.9%)	3.40 (1.01-11.46)	0.042	1.78 (0.32-9.91)	0.508
Admission	8 (11.8%)	60 (88.2%)	14.75 (1.36-15.59)	0.020	13.73 (3.01-62.59)	<0.001^∗^
Hospital stay >48 hr	7 (15.7%)	39 (84.7%)	3.41 (0.39-29.74)	0.261	NA	

^∗^Statistically significant at *P* < 0.05. AOR: adjusted odds ratio; COR: crude odds ratio; 1: reference group; 95% CI: 95% confidence interval; NA: not applicable.

**Table 4:** Antimicrobial susceptibility patterns of enterococci isolates from pediatric patients attending DRH from February to May 2019.

S. no.	Antibiotics	Susceptible (%)	Resistance (%)
1	Penicillin	6 (54.5)	5 (45.5)
2	Ampicillin	6 (54.5)	5 (45.5)
3	Vancomycin	6 (54.5)	5 (45.5)
4	Erythromycin	2 (33.7)	4 (66.7)
5	Ciprofloxacin	6 (54.5)	5 (45.5)
6	Nitrofurantoin	2 (33.4)	4 (66.7)
7	Tetracycline	1 (9.1)	10 (90.9)
8	Chloramphenicol	1 (16.3)	5 (83.7)
9	Amoxicillin/clavulanic acid	3 (27.3)	8 (72.7)

**Table 5:** Drug resistance pattern of enterococci isolates from pediatric patients attending DRH from February to May 2019.

Resistance rate	Combination of antibiotics	Number of isolates
R1	TET	2
R2	TET, AUG	2
R2	TET, VAN	1
R4	TET, P, AMP, AUG	1
R4	TET, VAN, AUG, CIP	1
R5	TET, P, AMP, E, CPR	1
R5	TET, P, AMP, VAN, CPR,	1
R8	TET, P, AMP, VAN, CPR, NIT, E, C	2
Total		11

E: erythromycin; TET: tetracycline; AMP: ampicillin; P: penicillin; AUG: amoxicillin/clavulanic acid; C: chloramphenicol; CIP: ciprofloxacin; VAN: vancomycin; NIT: nitrofurantoin; R1: resistance to one; R2: resistance to two; R4: resistance to four; R5: resistance to five; R8: resistance to eight drugs.

## Data Availability

Data supporting the conclusions of this article are within the manuscript.

## Abbreviations

APHI: Amhara Public Health Institute
ATCC: American Type Cell Culture
BEAA: Bile Esculin Azide agar
BHI: Brain heart infusion
CDC: Centers for Disease Control and Prevention
CLSI: Clinical and Laboratory Standard Institute
CSF: Cerebrospinal fluid
DRH: Dessie Referral Hospital
HAIs: Healthcare-associated infections
HIV: Human immunodeficiency virus
IPC: Infection prevention and control
MDR: Multidrug resistance
MHA: Muller Hilton agar
SOPs: Standard operating procedures
SPSS: Statistical Package for Social Science
UTI: Urinary tract infection
VRE: Vancomycin-resistant enterococci.
